# Efficient Smart CMOS Camera Based on FPGAs Oriented to Embedded Image Processing

**DOI:** 10.3390/s110302282

**Published:** 2011-02-24

**Authors:** Ignacio Bravo, Javier Baliñas, Alfredo Gardel, José L. Lázaro, Felipe Espinosa, Jorge García

**Affiliations:** Electronics Department, University of Alcala, Alcala de Henares, Madrid 28871, Spain; E-Mails: balinas@gmail.com (J.B.); alfredo@depeca.uah.es (A.G.); lazaro@depeca.uah.es (J.L.L.); felipe@depeca.uah.es (F.E.); jorge.garcia@depeca.uah.es (J.G.)

**Keywords:** FPGA, CMOS sensor, Ethernet, intelligent camera

## Abstract

This article describes an image processing system based on an intelligent ad-hoc camera, whose two principle elements are a high speed 1.2 megapixel Complementary Metal Oxide Semiconductor (CMOS) sensor and a Field Programmable Gate Array (FPGA). The latter is used to control the various sensor parameter configurations and, where desired, to receive and process the images captured by the CMOS sensor. The flexibility and versatility offered by the new FPGA families makes it possible to incorporate microprocessors into these reconfigurable devices, and these are normally used for highly sequential tasks unsuitable for parallelization in hardware. For the present study, we used a Xilinx XC4VFX12 FPGA, which contains an internal Power PC (PPC) microprocessor. In turn, this contains a standalone system which manages the FPGA image processing hardware and endows the system with multiple software options for processing the images captured by the CMOS sensor. The system also incorporates an Ethernet channel for sending processed and unprocessed images from the FPGA to a remote node. Consequently, it is possible to visualize and configure system operation and captured and/or processed images remotely.

## 1. Introduction

These days there are numerous situations where one or several machine vision cameras are routinely used to make daily life more comfortable. For example, they are used in door intercom [1] and video surveillance applications [2,3] and even in mobile phones [4]. In other situations, it is not only of interest to visualize images, but also to process them in order to extract certain information from the automatically captured scene, without the need for direct human intervention. An example would be video surveillance systems capable of detecting that someone is in the area under surveillance and notifying the police [5]. There is always a common element to all the above examples, and many others, namely the use of a sensor to capture the image to be visualized. Basically, there are two current alternatives: Charged Coupled Device (CCD) sensors and Complementary Metal Oxide Semiconductor (CMOS) sensors. The former have largely fallen into disuse, fundamentally due to the better features offered by CMOS technology as regards vision systems capable of image processing. Although CCD sensors provide better image quality than CMOS sensors, this is not the only factor to take into consideration when choosing a sensor, and CMOS sensors offer other features such as digital interface control, high speed exposure, functionality, *etc.*, which render them ideal in terms of the basic elements of an embedded image capture and processing system.

Consequently, an important contribution of this study is the construction of a specific image capture and processing platform on which to implement image processing techniques which are adaptable to different situations [6]. These kinds of platforms are generally known as intelligent cameras, and are characterized by integrating in one optical system a lighting system, an image capture system and an image processing system. Thus, the processing system endows these cameras with a level of intelligence that permits multiple applications to be implemented without the need for manual control. In the context of applications, advantages include autonomy, freeing up resources from the processing load and preventing communication bottlenecks. These advantages are conditioned by constraints of real-time operating cost, size and capacity.

Generally, intelligent camera hardware (HW) can be divided into three main blocks:

- Data acquisition: essentially comprising the image sensor, although it may include other devices used to complete the information required by the application (additional lighting, inertia sensors, GPS, microphones, *etc.*).
- Processing: processes the information specific to the application. The results obtained are usually sent to a host, trigger other processes or are used in a control loop.
- Communication interface: the interface through which the camera communicates with the exterior, with a host or connects to a shared resource.

As regards the processing devices, the most common are microcontrollers or microprocessors, Digital Signal Processors (DSPs), Field Programmable Gate Arrays (FPGAs) and multimedia processors. Application Specific Integrated Circuit (ASIC) devices, Single Instruction Multiple Data (SIMD) processors and embedded FPGA processors (soft/hard cores) are also used. The choice of which to use will depend on the application, and basic considerations include flexibility, processing performance, low Non-Recursive Engineering (NRE) cost, low energy consumption and level of programmability. This means that because the HW used is versatile, it can be used for different applications, adapting only the software (SW). The opposite situation would be systems based on ASIC devices whose functionality is only for which they were designed. If you want to reuse them for new applications, this will result in a higher NRE cost because they require at a minimum some new HW.

From among these options, FPGAs represent an excellent choice as regards high performance applications and low production level. Indeed, FPGAs have undergone remarkable technological development in recent years and their use has increased considerably in fields such as aeronautics, the military, industry and research. Due to the increasing number of logic elements per device, increased clock frequencies, and to the possibility of using massive parallel processing, FPGAs now come close to ASICs in terms of data processing tasks. Furthermore, if we consider the possibilities they offer of instantiating DSP blocks, embedded processors (soft/hard cores) and memory controllers, among other features, FPGAs constitute a very interesting device for implementing intelligent camera architecture.

The implementation of image processing algorithms on reconfigurable hardware permits an increase in algorithm efficiency. Previous studies such as [7] have demonstrated the advantages of hardware processing. In the study cited, detection of moving objects in a scene using Principal Component Analysis (PCA) techniques was entirely implemented on an FPGA. The high number of operations required by the PCA technique implies high execution times if conventional processing platforms, which are normally sequential, are used.

The bandwidth necessary for the communication interface is basically defined by whether complete-resolution images are to be sent, or only certain information. This bandwidth is also closely related to the image sensor and the number of images or pixels it is capable of capturing per second. Other factors to consider when choosing an interface include the communication media (cable or wireless), latency, response time and communication compatibility and protocol license. In this article, we present a solution aimed at integrating hardware and software processing in the same device, specifically, a SCVX4 camera (a smart camera based on Virtex 4). This represents the first version of an intelligent camera with an architecture based on a Xilinx Virtex 4 FPGA with embedded PowerPC 405 processor. The aim was to implement image processing applications mainly on reconfigurable hardware, that is, not only to carry out the classical hardware image pre-processing (gain correction and noise pattern correction, decimation/binning, compression, *etc.*), but also to implement processing algorithms capable of extracting more abstract information (PCA techniques, segmentation, classification, *etc.*). The system was completed with a PowerPC processor for implementing/managing mixed processing, which combined the execution of hardware processing blocks with software processing blocks. As regards image capture, we chose the Aptina (formerly Micron) MT9M413C36STM [8], which captures images in black and white at a maximum speed of 500 images per second, with a maximum spatial resolution of 1,280 × 1,024 pixels. The system proposed was validated at architectural level, optimizing the datapath and different pipelines for each design block. It was not the aim of this study to implement novel image processing, and accordingly, the camera implemented image processing based on singular value thresholding, which enabled us to validate the present proposal. With this system, thresholding can be implemented on the hardware or the software block in order to compare the different implementations at the same time as validating the intelligent camera architecture.

The remainder of the article is divided into the following sections: Section 2, which reviews some of the most important studies regarding FPGA image processing; Section 3, which presents the most relevant characteristics of CMOS and CCD sensors; Section 4, which details the structure of the design presented here; Section 5, which gives the most significant results obtained in this study, and lastly; Section 6, which summarizes the most important conclusions.

## 2. Related Studies

In many applications, real-time image processing requires rapid and continuous calculation of operations in order to run on a determined system. This is basically due to the high computational load associated with image processing algorithms. Recently, reconfigurable hardware devices have become an alternative for image processing due to their low cost and many features. However, this type of device presents considerable problems from the point of view of implementing algorithms, since the tools for their synthesis have not been developed as far as could be wished, nor have they been standardized to date.

Within the context of image processing with reconfigurable devices, an increasing number of studies have been carried out, since FPGAs attain high levels of performance within this area of research and development. However, the fact that the sequential execution of many image processing algorithms can be partitioned facilitates the design of different processing elements which can operate in parallel, accelerating execution time.

Since the appearance of FPGAs at the end of the 1980s, their use in image processing has increased steadily. Initially, the first studies of FPGA processing used a custom machine as the platform [9–11]. At the same time, other studies such as those reported in [12,13], began to use the characteristics of these devices in simple image pre-processing algorithms. A list of all the machines constructed with FPGAs from 1994 to 1999 is given in [14].

The technological development of FPGAs brought about the gradual disappearance of custom machines constructed using multiple FPGAs, in favour of a single FPGA operating as a coprocessor. These thus coexisted with a PC or a DSP, the latter handling the complex computational operations whilst the FPGA handled low level processing. Simple algorithms, such as filter or convolution algorithms, were the habitual applications used in these systems. Some examples of this type of operation are given in [15,16]. This type of system is still very much in use today. Examples of digital pre-processing techniques are given in [17,18].

The implementation of high level algorithm processing on FPGAs did not appear until the beginning of 2000 [19,20], and it was at this point when the new generation of FPGAs began to emerge, which included new features such as digital processing hardware units, cores and memory blocks.

As regards studies implementing intelligent cameras with technologies similar to that presented here, it is possible to find intelligent camera architectures implementing combinations of the various processing devices available on the market. For example, the WiCA camera, developed by the NXP research unit (formerly Philips) [21], uses up to two 300 Kpíxel VGA image sensors connected to a SIMD IC3D processor, with an ATMEL 8051 microcontroller to implement a ZigBee communication interface. This architecture is especially designed to achieve high performance low level image processing, where the same operation is applied to all pixels in the image. This camera is oriented to distributed processing applications, such as, for example, distributed face detection, where fusion of the detection results from various cameras is merged to reduce failure rates [22].

The high speed intelligent camera developed in the Le2i laboratories at the University of Burgundy [23] uses the same Aptina image sensor as that used in the present study. However, in this case, a Xilinx Virtex-II FPGA is used to pre-process images at a pixel level (Sobel filter, erosion/dilatation, calculating centre of mass, *etc.*), before transmitting via USB 2.0 interface.

The SeeMOS architecture presented by LASMEA [24] consists of a 4 Mpixel CMOS LUPA4000 monochrome image sensor with Cypress Semiconductors, an Altera Stratix FPGA complemented by a Texas Instruments TMS320C6455 DSP and a Fireware communication interface. It also includes various inertia sensors (three accelerometers and three gyroscopes) integrated into the same architecture. The SeeMOS camera is designed to provide ample image processing flexibility, and algorithms can be implemented on either software or hardware.

Other projects of interest, given their Open Source nature, include the intelligent CMU-cam camera, developed at Carnegie Mellon University, and the NC3x3 camera developed by Elphel. The CMU-cam project [25] is currently developing the third version (CMUcam3), using an Open Source vision platform consisting of CIF resolution (352 × 288 pixels) colour image sensor and a microcontroller based on an ARM7TDMI core.

In addition to comprising Open Source software (GNU/GPL license), Elphel’s NC3x3 project [26] also uses Open Source hardware. This intelligent camera consists of a modular platform (image sensor, processing and edge amplifier cards, *etc.*). The latest version released by Elphel is the NC353 camera, the architecture of which is based on a 5 Mpixel Aptina MT9P001 colour image sensor, a Xilinx Spartan III FPGA, a 32 bit ETRAX processor which runs on GNU/Linux and a 100 Mbit Ethernet communication interface. An additional card adds an external SATA port, internal and external USBs, an interface for synchronizing various cameras and/or sensors, and an internal IDE port. The Elphel camera is distributed with HDL modules for image pre-processing [Fixed Pattern Noise (FPN) elimination, RS compensation, *etc.*] and image compression (JP4, JP6, *etc.*) via implementation of an efficient pipeline which provides a continuous flow of images. Pre-processing and compression is carried out by the FPGA, which employs shared memory to send the images to the ETRAX processor for transmission via Ethernet.

There are many other OEM cameras developed by manufacturers all over the world: the VC44xx, developed by VC Vision Components, the NI 17xx from National Instruments, the IVC-2D and IVC-3D from SICK IVP and the XCI from Sony, to name but a few. The majority of these cameras are industrial, which implies that they are to a large extent closed source and inflexible in terms of implementing new processing algorithms, in addition to being very expensive.

The majority of the systems described here share in common the use of a device dedicated to pixel level image processing together with an external processor which executes high level software processing and serves as the communication interface. The capacity to implement efficient processing algorithms on hardware simplifies the software processing requirements. Thus, the implementation of these algorithms on reconfigurable hardware in symbiosis with an embedded processor in the same chip (hard/soft core) constitutes a system which eliminates the need for an external processor, since the same embedded processor can manage both purely sequential processing and the communication interface. In addition, internal FPGA resources allow hardware reconfiguration without the need to completely stop device applications.

## 3. CCD *vs.* CMOS

As regards cameras and vision systems, the image sensor plays a crucial role in system performance. Although image quality (sensitivity, dynamic range) and resolution (pixel number) are important parameters which characterize the image sensor, other considerations include pixel rate (number of pixels or images per second), addressing modes (sampling modes, random access), ease of integration into the system, and the logic necessary to control acquisition.

CCD and CMOS are the two most frequently used technologies in image sensors today [27,28]. CCD sensors implement readout techniques based on displacement maps: the electron charge accumulated by each photodiode from one row (pixel) is displaced to the next in the same column until the last photodiode in the same, which is connected to an analog converter and data acquisition system. Control electronics system permit synchronized acquisition of values for the entire sensor matrix. There are three types of CCDs, defined by the readout technique used:

1. Full frame. The pixel matrix is formed entirely by photodiodes. The sensor takes exposures via a mechanical shutter release, and it is necessary to displace the entire matrix to the data acquisition electronics.
2. Frame Transfer. Half of the matrix is covered by an opaque film. Shutter release in this case is electronic, and the charge from all the photodiodes is simultaneously displaced to the opaque area for subsequent readout. This type of CCD achieves lower exposure times and reduces luminous sensitivity.
3. Interline CCD. The matrix includes intermediate storage devices in each column and row, which avoids noise accumulation due to charge displacement and permits rapid readout. Due to the type of construction, these CCD sensors suffer from a problem known as Blooming. If one of the photodiodes is overexposed, this triggers spontaneous charge displacement, affecting all the other photodiodes in the same column. Some CCDs implement fabrication techniques which mitigate the Blooming effect.

CMOS sensors use the latter technology to implement a photosensitive matrix. They are based on digital memory readout techniques, using row decoders which permit random access and the selection of regions or windows of interest. Each pixel contains the electronic circuitry necessary to convert the electrical charge into tension, and this affects the fill factor which relates the data acquisition area to the pixel matrix conversion area. Thus, CMOS sensors usually have a fill factor of around 50% compared to the factor of around 100% which CCD sensors can reach. Due to their architecture, these sensors attain very low readout times and consequently, in order to characterize the image acquisition task, pixel number per second is used rather than the image number per second used by CCD sensors. Thus, CMOS sensors are characterized by obtaining high pixel per second rates. In addition, by selecting small pixel subregions, it is possible to obtain image per second rates which are above the nominal rate for the entire matrix. Other characteristic advantages of CMOS sensors include a high dynamic range, ease of integration and low Blooming effect. However, a disadvantage is that the image performance of CMOS sensors is low compared to CCD sensors, mainly due to Fixed Noise Pattern (FNP). This noise is the consequence of uncertainty in the electrical characteristics of the electronic circuitry associated with each pixel and each column output amplifier, resulting in an image presenting static offset. However, the FNP can be eliminated using digital processing, and with CMOS technology, additional hardware can be integrated into the same chip for this filtering process. Many of the current CMOS sensors include additional logic which adds more complex readouts (inverted or rotated images), communication interfaces (I2C), or even prior image processing (decimation, binning).

Cameras employed for vision applications most frequently use CMOS sensors. The lower image resolution and performance of these sensors is considered almost irrelevant compared to their image per second rate, selective acquisition of areas of interest, ease of integration and additional features provided by hardware embedded in the sensor. Furthermore, given that the images produced by this type of system will be digitally processed, the need for processing to eliminate FNP noise implies a negligible computational load.

## 4. APTIMA CMOS Sensors

The camera presented in this article uses the monochrome version of the Aptina MT9M413C36STM sensor [8]. This is a 1.3 Mpixel CMOS sensor capable of producing 500 images per second at maximum resolution (1,280 × 1,024 pixels). Other characteristics of interest include the possibility of windowing, enabling a minimum region of 1,280 × 1 pixels to be selected in order to achieve a speed of 4,000 fps, a 100 bit digital output bus (ten 10-bit pixels each), maximum data volume of 660 Mbps with a 66 MHz clock and an energy consumption of 300 mW at 300 fps (less than 150 mW at 60 fps).

This sensor was chosen primarily because of its high speed image capture performance, thus the final rate of the system (the camera) is defined by hardware processing efficiency. Another interesting advantage of this system from the point of view of handling is the possibility of exporting the Hardware Description Language (HDL) controller implemented on the FPGA to other Aptina models, since the sensors made by this company are highly compatible. This is the case of the Aptina MT9P001sensor, for example, implemented on the Elphel NC353camera. The basic characteristics of this latter include 5 Mpixel (2.592 H × 1.944 V) resolution, sensitivity to the entire visible spectrum and an image per second rate of up to 15 fps (70 fps for VGA resolution). Despite being a CMOS sensor, its low noise and high sensitivity mean that it is possible to obtain images of a quality comparable to that of CCD sensors at a low cost and with the ease of integration provided by CMOS technology.

Returning to the MT9M413C36STM sensor, this offers maximum response to the visible spectrum and reasonable sensitivity to the infrared wavelength, thus making it especially useful for certain applications (such as night vision, for example).

The sensor’s address system is partial, only permitting rows of interest to be selected, not columns, and it uses a 10-bit address bus for this function. With this system, it is possible to read specific rows whilst ignoring others, which increases the number of frames per second when only a certain area of the image is of interest.

Two shutter release mechanisms are possible, Rolling Shutter (RS) or Global Shutter (GS). The first executes exposures row by row, whilst the second executes exposure of the entire matrix at the same time. With the RS method, higher image per second rates are achieved than with the GS method, but it also has the disadvantage of a time lag between rows which produces distortions to the image if the scene is not static. In turn, the GS system offers three different shutter and acquisition modes:

- Simultaneous Mode: frame exposure is carried out whilst the previous frame is being read. This mode increases the image per second rate.
- Sequential Mode: this mode also produces continuous video, but the exposure of an image only begins once the previous image has been read, producing lower speeds than the simultaneous mode.
- Single Frame: in this mode, images are acquired at the end of the process, and all exposures must be executed before the image can be read.

On the basis of the characteristics described above, the sensor chosen made it possible to cover a very wide variety of applications with requirements that could range from high image capture speed to high resolution.

## 5. New Architecture Proposal to Capture/Process Images

The architecture proposed is an intrinsic part of an FPGA, specifically, a Virtex 4 FX (XC4VFX12) with internal implementation of an embedded system, consisting of a PowerPC (PPC) 405 processor integrated into the same chip (hard core). In addition to PPC, this FPGA has mainly the following internal resources:

- Cell Logic Block (CLB), which implements the sequential and combinational logic functions. A CLB is composed by several slices where each slice contains Look Up Tables (LUT), multiplexers and Flip-Flop registers.
- Input-Output Block (IOB). These elements are related to the input/output terminals of the FPGA.
- Digital Clock Management (DCM). Thanks to this one, an efficient clock control can be done.
- Block RAM (BRAM): These blocks work as internal RAM of the FPGA
- Hardware Multipliers: Multiply operation is frequently used in many applications. Due to this, hardware multipliers are available inside of FPGA in order to perform this operation as quickly as possible.

**Figure 1.**
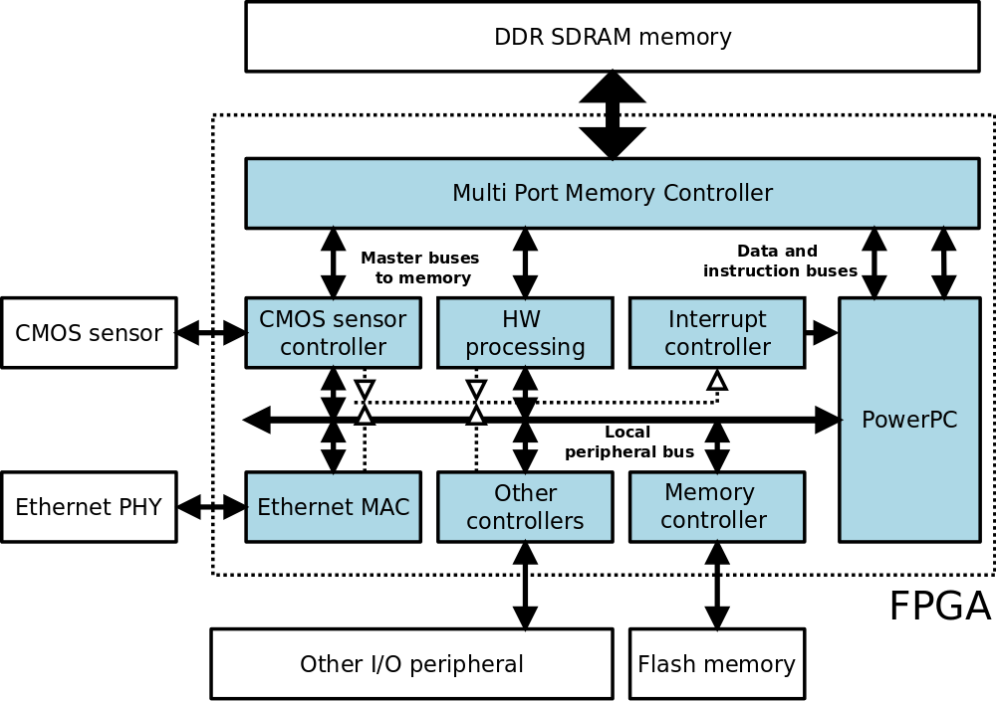
Architecture of Intelligent Camera based on FPGA.

The FPGA is connected to the Aptina MT9M413C36STM CMOS sensor, 32 Mb of DDR SDRAM memory, 8 Mb of Flash memory, a 100 Mbit Ethernet transceiver and other I/O peripherals such as an RS-232 port, LEDS and switches. Figure 1 shows a simplified block diagram of the architecture. The elements that make up this architecture are sufficient to successfully perform image processing operations. The DDR memory processes the images captured by the CMOS sensor and shares them with the PPC. This DDR memory acts as a video buffer for the image acquisition system, and as the system memory and RAM for the PPC. The Flash memory stores the programme to be executed by the PPC, which is initially downloaded via a bootloader to the DDR memory. An Ethernet port and the RS-232 port comprise the communication interfaces.

As regards the FPGA logic design (light blue area in Figure 1), all the internal blocks are implemented on reconfigurable logic except for the PPC processor, which is implemented on a silicon chip in the form of a mask-level chip layout (hard core). Management and control of the DDR memory is carried out by a multi-port controller (Xilinx MPMC core), which permits shared use of its entire address range or partitioning into various independent banks. This controller is necessary since, as can be seen in Figure 1, various modules within the system make use of the external DDR memory. In this way, the MPMC controller arbitrates memory access among the different modules, optimizing the execution of applications and not penalizing execution of the programme on PPC or on the other modules. The remaining logic blocks (controllers and peripherals) are connected to and controlled by the PPC through a local bus. In addition, some blocks are mapped onto the PPC interrupt lines by an interrupt controller. There are still further blocks which operate as clock generators, JTAG and bus arbitrators, among other functions, which are necessary for the correct operation of the system.

The HW processing block and the CMOS sensor controller in Figure 1 have an additional, second interface which provides them with direct access to the memory, and each is connected to a memory control port. The multi-port memory controller has a FIFO buffer for each channel which, together with the configurable arbitration mechanism, facilitates pseudo-simultaneous access to the memory from different ports. Memory access can be random or burst access of up to 4,096 bytes.

**Figure 2.**
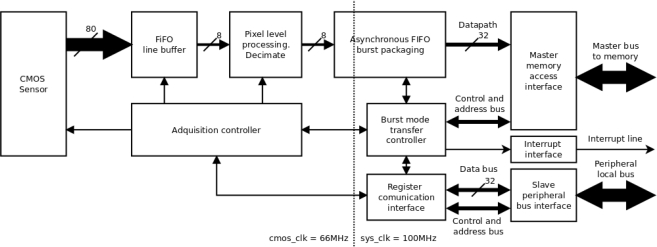
FPGA Block diagram of Capturing and Controlling CMOS Sensor.

Of the different blocks which configure the design in Figure 1, the CMOS sensor controller is the most important, since it enables adjustment of the internal parameters of the sensor from the FPGA (Figure 2). In this way it is possible to adjust exposure time from 0 to 300 ms, perform configurable windowing and adjust resolution via decimation of up to an order of 10 on the CMOS sensor resolution (1,280 × 1,024). The CMOS sensor supports both RS and GS shutter release mechanisms in the Sequential and Single Frame modes, and these parameters are also configurable for the local bus system through the register interface implemented within the FPGA CMOS controller.

The internal structure logic designed for the CMOS controller (see Figure 2) is managed by two clock domains, one of 66 MHz (clock signal frequency imposed by the FPGA on the CMOS sensor) synchronized with image acquisition, and another of 100 MHz (the FPGA master clock signal frequency), synchronized with the system buses. An asynchronous First In First Out (FIFO) memory with asymmetric datapath width ensures compatibility between both clock domains and adapts datapath width between the image acquisition bus and the system bus. The FIFO is used to generate data bursts in block data memories of 512 32-bit words.

In each clock cycle, the CMOS sensor delivers 10 10-bit encoded pixels to the FPGA, received by the FPGA from the asymmetric FIFO, which implements a line buffer (1,280 pixels). The input FIFO data are truncated to 8 bit in order to optimize the access to external memory. Image size can be reduced by up to ten during output by a decimator. Subsequently, the data are packaged and transferred to memory in burst mode.

An acquisition controller manages the FIFO line memory, decimation and the CMOS sensor control bus, implementing the different image acquisition modes mentioned previously, which are parameterized by the register interface. Burst mode transfer of the image to memory is also parameterized by register interfaces. Using an interrupt line, the CMOS controller indicates when the writing burst to memory has finished and a complete frame has been written.

The asymmetric and asynchronous FIFO memory has a modular, isolated design which facilitates use of another CMOS sensor module, for example the 5 Mpixel Aptina MT9P001 sensor. Integrating a new image sensor merely requires specification of the “Acquisition Controller” block specific to the sensor, adapting the datapath width via the FIFO line memory to the work frequency of the new sensor.

**Figure 3.**
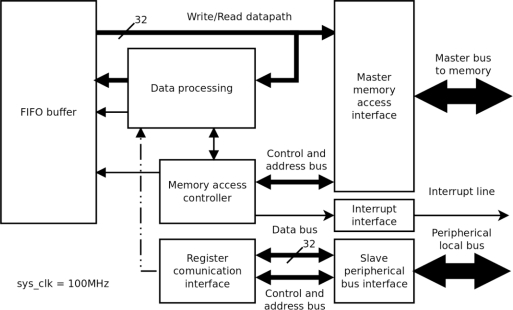
Internal Block of Hardware Processing Module.

The internal logic of the HW processing block in Figure 1 is analogous to that of the CMOS controller: a direct memory access read/write bus, an interrupt line and connection to the local peripheral bus (Figure 3). The input datapath is connected with the data processing block, which is parameterized by the register interface. The data are processed in blocks of 512 pixels and stored in a FIFO buffer. Data processing is synchronized by the memory access controller and once a pixel block has been processed, it is overwritten in the memory. The interrupt interface indicates when processing of a pixel block has terminated.

All the logic elements forming part of the design implemented on the FPGA are concurrent due to the hybrid pipeline system that combines the hardware and software domains. System execution flow can be divided into the following stages:

1. *Writing image into memory stage:* images captured by the CMOS sensor are read by the dedicated controller, pre-processed at bit level (decimated) and subsequently packaged in discrete pixel blocks and directly transferred in burst mode to the memory.
2. *Hardware processing of image stage:* once the first block of pixels has been transferred to the memory, an interrupt to the PPC processor, which manages the hardware processing of that pixel block, is generated.
3. *Software processing of image stage:* once hardware processing is completed, the corresponding block generates an interrupt which triggers software processing.
4. *Sending processing results stage:* Lastly, the information obtained from the scene or image after processing can be sent via Ethernet.

In order to simplify analysis and validation of the system, execution of image processing is sequential: the hardware processing is executed first, and then the software processing. As regards executing a specific processing algorithm, the hardware and software processing phases can be executed conditionally and/or alternately if necessary to optimize algorithm execution time.

**Figure 4.**
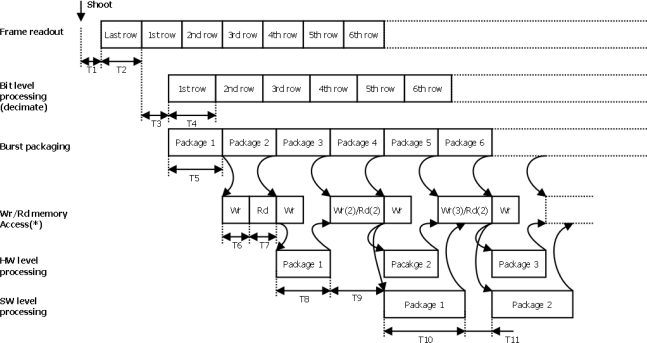
Internal Pipeline executing image capture and processing.

Figure 4 shows the scheduling framework of the pipeline for the execution flow stages mentioned previously. The schedule diagram starts with the capture of a new image in RS mode, and considers a null exposure time. The times TI to TII (see Table 1) associated with each stage provide the image per second rate (fps) at a given point in the pipeline or at the end of a specific stage. It should be noted that the simultaneous access of different hardware blocks to the memory implies a bottleneck which results in pipeline stalls between pipeline stages. This is due to the fact that the multi-port memory controller has one FIFO buffer per port and does not ensure immediate memory access. Thus, non-deterministic latencies exist associated with each memory access, reducing the image per second rate of the system. In order to reduce pipeline latencies or stalls, an analysis was carried out of the period of memory access available to each block involved in the pipeline, to determine and set priorities for each of the memory controller ports.

The total image per second rate the system is capable of achieving is obtained from a partial analysis of each stage in execution flow. Thus, for each stage an fps rate is defined or, what is the same, the execution period of each stage:

1. T_FRAME_MEM_: period of the stage where image is written into memory.
2. T_FRAME_HW_: period of the hardware processing stage.
3. T_FRAME_SW_: period of the software processing stage.
4. T_SEND_: period associated with the stage of transmitting image processing results by Ethernet.

When calculating the total frames per second rate, in addition to considering periods associated with execution stages, sensor exposure time should also be taken into account. Where this exists, the total frame per second rate is given by Equation (1):

(1) $$\mathrm{Frame\_rate}=\frac{1}{T_{\mathrm{EXP}}+\mathrm{MAX}\{T_{\mathrm{FRAME\_MEM}},T_{\mathrm{FRAME\_HW}},T_{\mathrm{FRAME\_SW}},T_{\mathrm{SEND}}\}}$$

The period T_FRAME_MEM_ is given by Equation (2). As can be observed from Figure 4, this period is principally due to time T_5_, the time necessary to package a pixel burst in the controller’s FIFO buffer. The expression includes the possible latency in memory write access Δ*T*6:

(2) $$T_{\mathrm{FRAME\_MEM}}=T_{1}+T_{2}+T_{3}+\left(\frac{\frac{{\mathrm{SIZE}}_{\mathrm{COL}}\cdot {\mathrm{SIZE}}_{\mathrm{ROW}}}{D^{2}}}{{\mathrm{SIZE}}_{\mathrm{BURST}}\cdot N}\right)\cdot T_{5}+T_{6}+\sum_{<\mathrm{frame}>}\Delta T6$$

For the rate obtained following the hardware processing represented by Equation (3), the period associated with T_FRAME_HW_ is principally due to the size of one pixel burst and the time taken to process it, T_8_, this time depending on the processing algorithm implemented.. Note that the hardware processing datapath is 32 bits or 4 pixels, and high hardware processing performance is obtained by implementing parallel data processing algorithms. Memory access time is represented by the sum of time, T_9_, consisting of the system’s read/write access times and the associated latencies:

(3) $$T_{\mathrm{FRAME\_HW}}=T_{1}+T_{2}+T_{3}+T_{5}+T_{6}+T_{7}+\left(\frac{\frac{{\mathrm{SIZE}}_{\mathrm{COL}}\cdot {\mathrm{SIZE}}_{\mathrm{ROW}}}{D^{2}}}{{\mathrm{SIZE}}_{\mathrm{BURST}}\cdot N}\right)\cdot T_{8}+\sum_{<\mathrm{frame}>}T_{9}$$

As regards the T_FRAME_SW_ period of (1) associated with the software processing rate, this mainly depends on algorithm execution time T_10_ and, as in previous stages, on latencies due to system memory or Cache access represented by the sum of T_11_:

(4) $$T_{\mathrm{FRAME\_SW}}=T_{1}+T_{2}+T_{3}+T_{5}+T_{6}+T_{7}+T_{8}+T_{9}+\left(\frac{\frac{{\mathrm{SIZE}}_{\mathrm{COL}}\cdot {\mathrm{SIZE}}_{\mathrm{ROW}}}{D^{2}}}{{\mathrm{SIZE}}_{\mathrm{BURST}}\cdot N}\right)\cdot T_{10}+\sum_{<\mathrm{frame}>}T_{11}$$

Finally, as regards data transmission time, the bandwidth is defined by the interface and communication protocol used—in this case, Ethernet 100 Mbps and TCP/IP. The T_SEND_ period depends, in the last instance, on the quality of data to be sent. Given that the system is oriented to internal processing, the quantity of data to transmit is low compared to the quantity of data needed to transmit a complete image. Furthermore, transmission of a complete processed image is only necessary during validation and refinement of the processing algorithm, which does not require real time image transmission. Thus, the data transmission time T_SEND_ does not represent a limitation on the execution flow of the system.

## 6. Results

The system for capture and processing of images using a CMOS sensor controlled by an FPGA described above was validated under the following conditions:

- Pixel burst size of 512 words for the CMOS Sensor Controller and 128 words for the processing HW system.
- The algorithms implemented for hard- and software processing were thresholding. This has made it possible to compare the two implementation domains.

The FPGA model employed was the Xilinx XC4VFX12 0, using the ISE, EDK and SDK Xilinx tools, version 10.1. Table 2 gives a summary of the resources consumed in the FPGA.

The present system is limited by the number of occupied slices (almost all are in use), and it is important to note that the FPGA used has the lowest number of slices of all the FX family (5,472 slices compared to 63,128 slices in the XC4FX140 chip). Therefore, in order to implement more complex image processing algorithms it would be necessary to choose a superior FPGA model.

**Figure 5.**
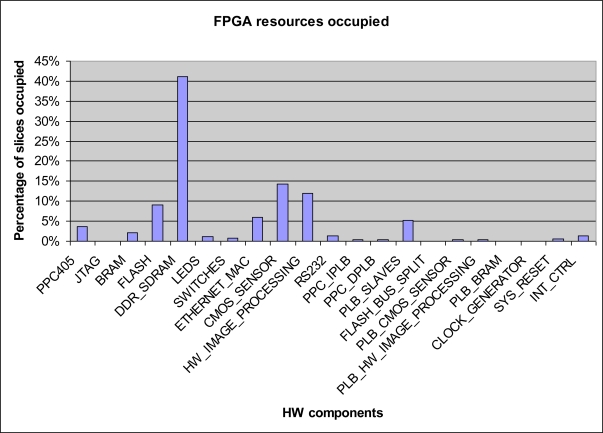
Internal FPGA resources occupied by each module of Figure 1.

An analysis of the number of slices occupied in the different components of the system is given in Figure 5, showing a comparative diagram of distribution of occupied slices in the system. It can be seen that the component which requires most slices is the DDR memory controller, with a 40% occupation rate, followed by the CMOS controller with an occupation rate of around 15% and the hardware processing block, occupying around 12% of slices.

As regards timing and frequency constraints, Table 3 shows the results obtained from the implementation tool following synthesis. It can be seen that all the modules used were well within the imposed constraints.

**Figure 6.**
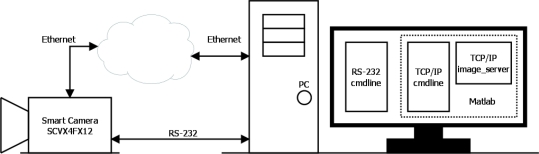
System used to validate the intelligent camera based on FPGAs.

As for the actual tests used, the bank of tests employed to validate the camera is given in Figure 6. It can be observed that the camera is connected to a PC via a serial RS-232 connection and LAN Ethernet. This is because the camera implements a serial port command interface for test tasks and configuration, and a TCP/IP socket, which in addition to the serial port functions, receives and transmits the original or processed images.

**Figure 7.**
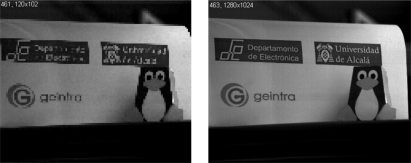
Captured and processed image from FPGA with different resolution. On the left, an 120 × 102 pixel image and on the right, a 1,280 × 1,024 pixel image.

It is possible to configure the various camera parameters (resolution, decimation factor, RS mode and exposure time), refine operation at application level and control image acquisition and processing via the command interface. Images are sent using an image server. Figure 7 shows two real images at different resolutions captured using the camera.

**Figure 8.**
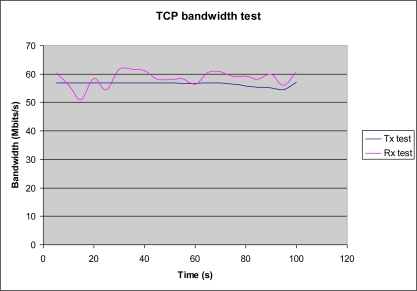
Experimental TCP Bandwidth Test with Ethernet FPGA IP Core.

Ethernet bandwidth was tested using the Iperf tool, which measures maximum bandwidth performance for TCP and UDP connections. Transmission and reception was sampled at 5 seconds intervals over a 100 seconds period. Given the fact that microprocessor is working in several tasks (included Ethernet data transmission) the real Ethernet bandwidth decreases until 60 Mbpps. This situation can be checked in Figure 8, where reception is more irregular than transmission behaviour. In any case, the results given in Figure 8 show that both transmission and reception bandwidth satisfied system requirements and the achieved bandwidth is close to maximum rate for Ethernet Phy. device (up 100 Mbps).

Frame rate is one of the most typical index to validate the efficacy of an image processing system. Due to this fact, the architecture implemented has been tested with four different tests which are continuously running, in order to extract different frame rates:

- Test 1 (Capture): In this one a simple image capture and loading has been done by the FPGA. Thanks to this test the achieved frame rate will be the best case because no HW or SW processing is done.
- Test 2 (Capture + SW processing): This situation emulates an application in which the HW FPGA elements are only used for image capture and the microprocessor (SW stage) runs a SW image processing application (in our case Thresholding).
- Test 3 (Capture + HW processing): In this case, internal HW FPGA resources are also used to implement an image processing algorithm (Thresholding). The Microprocessor activity is reduced to manage and control the different HW enable signals and Ethernet Transmission.
- Test 4 (Capture + SW processing + HW processing): This situation will be the best case to apply a FPGA as a smart camera. All internal resources (HW and SW) are ready for image capture and processing application. Thus, HW FPGA internal resources are in charge to capture and processing image and the Microprocessor also implements a image processing algorithm. In HW and SW cases, a Thresholding application has been used.

**Figure 9.**
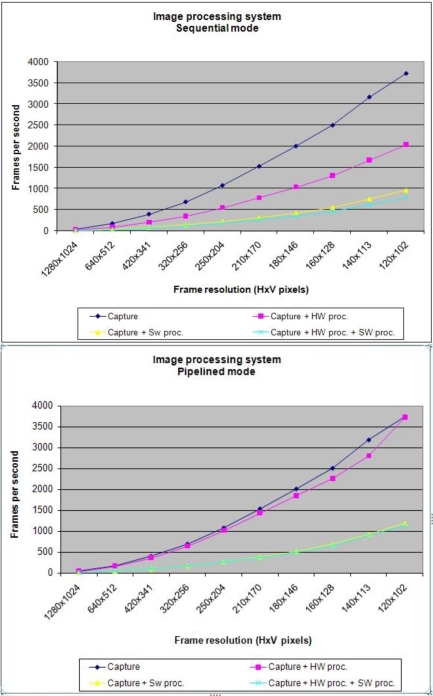
Frame rates for a sequential (upper) and pipelined mode (lower).

All previous tests have been launched for two different modes: First one is a complete sequential system in which no parallel process (pipelined) has been implemented. Second one is the opposite, a system with the maximum parallel level with the capacity to develop. Figure 9 shows the results for the four different tests in both modes (sequential and pipelined) with different image sizes.

**Figure 10.**
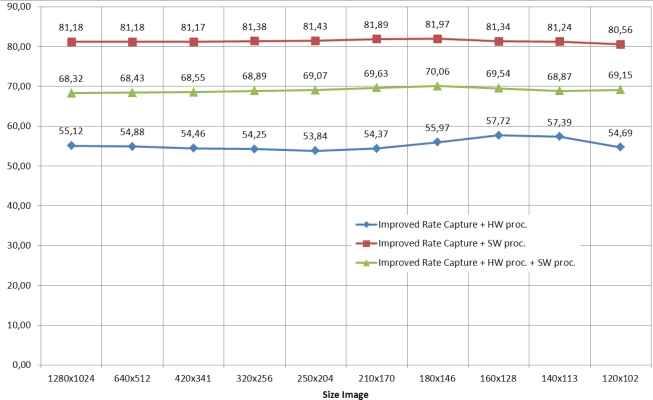
Improved pipelined *vs.* sequential frame rate.

The number of frames per second achieved by the parallel mode is higher for all modes presented in Figure 9. According to the information in this figure, it has been generated Figure 10 which shows the percentage improvement in parallel mode.

It can be verified the degree of improvement is fairly constant in all cases. This situation is due to the improvement does not depend on the size of the image. If it is greater, the system with parallel mode is at full capacity after an initial latency. In the situations shown in Figure 10, it is important to highlight the results achieved in “Improved Rate Capture and Processing Software”. The percentage improvement in parallel improves at less 80% than sequentially. This is because through the parallel mode, while an image is processed simultaneously a new image is captured.

## 7. Conclusions

This article presents the architecture for image capture and processing using a high speed CMOS sensor implemented on an FPGA. In the present proposal, a versatile hardware/software system was implemented on the above platform, which enabled us to develop an intelligent camera based on an FPGA, capable of internal image processing without need to use an external PC. The existence of a embedded microprocessor in the FPGA provides the system with the flexibility to choose which parts of an image processing algorithm to implement on the software (programme housed in the microprocessor) or hardware (using the HDL modules which are encapsulated as microprocessor peripherals): Thus, and with the aim of parallelizing tasks in order to attain concurrent execution, it is possible to attain high speed image processing. In order to obtain the highest fps rate possible, an exhaustive and in-depth analysis was carried out of the time consumed by each FPGA internal logic block.

Due to the hardware features of the architecture developed here, an Ethernet channel is available, providing the system with the possibility of remote configuration of the CMOS sensor’s internal parameters and other parameters of the image to be processed. Furthermore, and due to this possibility, images can be sent to a remote location with or without FPGA processing. The present study also included an in-depth test of the Xilinx Ethernet controller function, demonstrating that with a real-life platform and a specific application, the maximum transmission and reception rates supplied by Xilinx are not viable.

The use of Aptina CMOS sensors, and consequently, the use of HDL modules to control them, means that these can be reused in other sensor models without requiring more than minor modifications to the control and data buses connecting the FPGA and the CMOS sensor.

## Figures and Tables

**Table 1.** Times associated with the Internal Pipeline of Figure 4.

**Time (s)**	**Description**

*T*_1_ = 17 ·*T_CLK_CMOS_*	Latency from giving the signal to shoot a new image with null exposure time.
*T*_2_ = 134 · *T_CLK_CMOS_*	Time to write one row in FIFO buffer.
*T*_3_ = 4 ·*T_CLK_CMOS_*	Latency before decimation.
$T_{4}=\left(\frac{\mathrm{ROW\_SIZE}}{D}\right)\cdot T_{\mathrm{CLK\_CMOS}}$	Time to decimate one row
*T*_5_ = (*BURST_SIZE* + 23) · *T_CLK_CMOS_*	Time to package one burst in FIFO buffer.
*T*_6_ = ((*BURST_SIZE* + 255) · *T_CLK_SYS_*) + Δ*T*6	Time to write one pixel burst into memory.
*T*_7_ = ((*BURST_SIZE* + 255) · *T_CLK_SYS_*) + Δ*T7*	Time to read one pixel burst in memory
*T*_8_ = *f* (*hw_proc*)	HW processing time of one pixel burst.
*T*_9_	Memory access time between HW processing of pixel bursts. Includes memory access latency.

*T*_10_ = *f* (*sw_proc*)	SW processing time of one pixel burst loaded in Cache Memory.

*T*_11_	Memory access time to update one pixel burst for SW processing in Cache Memory

where:
T_CLK_CMOS:_ CMOS controller clock signal period (f_CLK_CMOS_ = 66 MHz). T_CLK_SYS_: clock signal period of the architecture’s internal buses (f_CLK_SYS_ = 100 MHz). ROW_SIZE: number of pixels per column in the CMOS sensor (1,280 pixels). COL_SIZE: number of pixels per row in the CMOS sensor (1,024 pixels). D: decimation factor (1 to 10). BURST_SIZE: number of words for access memory with a pixel burst. N: number of bytes per word of the memory data write bus (4 bytes/word). Δ*T*6: memory write latency. Δ*T*7: memory read latency.

**Table 2.** Summary of internal FPGA resources used.

Number of DCMs	1 out of 4	25%
Number of External IOBs	242 out of 320	75%
Number of PPC405_ADVs	1 out of 1	100%
Number of RAMB16s	28 out of 36	77%
Number of Slices	5,470 out of 5,472	99%
Number of SLICEMs	395 out of 2,736	14%

**Table 3.** User timing constraints for the developed design.

**HW Component**	**Clock**	**Maximum Frequency (MHz)**	**Required Frequency (MHz)**
PPC405	DPLB0_PLB_Clk DPLB1_PLB_Clk IPLB0_PLB_Clk	209.266 346.663 287.965	100 100 100
	IPLB1_PLB_Clk	287.965	100
PLB_SLAVES	PLB_Clk	258.522	100
BRAM	BRAM_Clk	250.240	100
RS232	SPLB_Clk	218.948	100
LEDS	SPLB_Clk	343.536	100
SWITCHES	SPLB_Clk	343.536	100
FLASH	MCH_PLB_Clk	174.497	100
INT_CTRL	SPLB_Clk	265.555	100
DDR_SDRAM	PLB_Clk	369.399	100
PLB_CMOS_SENSOR	sysclk	150.115	60
	MPLB_Clk	195.792	100
	SPLB_Clk	151.357	100
ETHERNET_MAC	SPLB_Clk	149.157	100
	PHY_tx_clk	396.873	100
	PHY_rx_clk	305.446	100
PLB_HW_IMAGE_PROCESSING	SPLB_Clk	156.777	100
	MPLB_Clk	200.906	100

## References

[1] Shi Y, Tsui T (2007). An FPGA-Based Smart Camera for Gesture Recognition in HCI Applications. Lect. Note. Comput. Sci.

[2] Haritaoglu I, Harwood D, Davis L Multiple People Detection and Tracking Using Silhouettes.

[3] Mustafah YM, Bigdeli A, Azman AW, Lovell BC Smart Cameras Enabling Automated Face Recognition in the Crowd for Intelligent Surveillance System.

[4] Tierno J, Campo C (2005). Smart Camera Phones: Limits and Applications. IEEE Pervas. Comput.

[5] Tian G, Li J, Chen B A New Mobile Police Spatial Information Service Grid Computing Model Based on Mobile Agent.

[6] Bravo I, Jiménez P, Mazo M, Lázaro JL, Martín E (2006). Architecture Based on FPGA’s for Real-Time Image Processing. Lect. Note. Comput. Sci.

[7] Bravo I, Mazo M, Lázaro JL, Gardel A, Jiménez P, Pizarro D (2010). Intelligent Architecture Based on FPGAs Designed to Detect Moving Objects by Using PCA. Sensors.

[8] (2004). Datasheets: 1.3-Megapixel CMOS Active-Pixel Digital Image Sensor MT9M413C36STM. http://www.micron.com/products/partdetail?part=MT9M413C36STM.

[9] Arnold JM, Buell DA, Davis EG SPLASH 2.

[10] Ziegler H, So B, Hall M, Diniz PC Coarse-Grain Pipelining on Multiple FPGA Architectures.

[11] Piacentino MR, van der Wal GS, Hansen MW Reconfigurable Elements for a Video Pipeline Processor.

[12] Hamid G An FPGA-Bases Coprocessor for Image Processing.

[13] Wiatr K Pipeline Architecture of Specialized Reconfigurable Processors in FPGA Structures for Real Time Image Pre-Processing.

[14] Guccione S, Hartenstein R Alphabetical List of Reconfigurable Computing Architectures. http://www.site.uottawa.ca/~rabielmo/personal/rc.html.

[15] Dawood AS, Visser SJ, Willianms JA Reconfigurable FPGAs for Real Time Processing in Space.

[16] Draper BA, Beveridge JR, Willem Böhm AP, Ross C, Chawathe M (2003). Accelerated Image Processing on FPGAs. IEEE Trans. Image Process.

[17] Arias-Estrada M, Rodríguez-Palacios E An FPGA Co-Processor for Real-Time Visual Tracking.

[18] Meribout M, Nakanishi M, Ogura T (2002). A Parallel Algorithm for Real-Time Object Recognition. J. Patt. Recog.

[19] Martín JL, Zuloaga A, Cuadrado C, Lázaro J, Bidarte U (2005). Hardware Implementation of Optical Flow Constraint Equation Using FPGAs. Comput. Vision Image Understand.

[20] Díaz J (2006). Multimodal Bio-Inspired Vision System. High Performance Motion and Stereo Processing Architecture.

[21] Kleihorst R, Schueler B, Danilin A, Heijligers M Smart Camera Mote with High Performance Vision System.

[22] Jeanne V, Jegaden FX, Kleihorst R, Danilinand A, Schueler B Real-Time Face Detectionona Dual-Sensor Smart Camera Using Smooth-Edges Technique.

[23] Mosqueron R, Dubois J, Paindavoine M (2007). High-Speed Smart Camera with High Resolution. J Embed Syst.

[24] Dias Real F, Berry F, Marmoiton F, Serot J Hardware, Design and Implementation Issues on a FPGA Based Smart Camera.

[25] Rowe A, Goode A, Goel D, Nourbakhsh I (2007). CMUcam3: An Open Programmable Embedded Vision Sensor.

[26] Filippov A High Resolution Network Camera.

[27] Kölsch M, Butner S (2009). Hardware Considerations for Embedded Vision Systems. Adv Patt Recog Ser.

[28] Dias Real F, Berry F (2010). Smart Cameras: Technologies and Applications. Smart Cameras.

[29] Xilinx Company (2010). Virtex 4 Family Overview. http://www.xilinx.com/support/documentation/data_sheets/ds112.pdf/.

